# Manifestation and Markings of HIV Stigma in Indonesia: A Scoping Review

**DOI:** 10.3390/ijerph22060840

**Published:** 2025-05-27

**Authors:** Ni Kadek Sudastri, Luh Putu Lila Wulandari, Pande Putu Januraga

**Affiliations:** 1 Doctoral Program in Medicine, Faculty of Medicine, Udayana University, Denpasar 80232, Indonesia; hanyadastri@gmail.com; 2 PUI PT Center for Public Health Innovation (CPHI), Udayana University, Denpasar 80232, Indonesia; 3 The Kirby Institute, University of New South Wales, Sydney, NSW 2052, Australia; lwulandari@kirby.unsw.edu.au; 4 Centre for Adolescent Health, Murdoch Children’s Research Institute, Parkville, VIC 3052, Australia; 5 Department of Public Health and Preventive Medicine, Faculty of Medicine, Udayana University, Denpasar 80232, Indonesia

**Keywords:** stigma, discrimination, HIV, AIDS, Indonesia

## Abstract

Stigma related to HIV hinders access to healthcare services and worsens the quality of life for people living with HIV (PLHIV). A comprehensive understanding of HIV stigma is crucial for designing effective stigma interventions. This scoping review offers an overview of the manifestations and markings of HIV stigma in Indonesia. Articles published in PubMed, Scopus, Google Scholar, and other sources were searched. Studies selected were published between 2019 and 2023, written in English, and focused on the manifestations or markings of HIV stigma in Indonesia. A thematic approach was applied to analyze the findings. From an initial pool of 4776 articles, 745 advanced to the title and abstract screening process, with 40 ultimately included in the review. The findings indicate that HIV stigma in Indonesia manifested as avoidance of contact with PLHIV, differential treatment of PLHIV, negative reactions toward PLHIV, and self-stigma. These stigmatizing behaviors were observed across various societal levels, including family members, the general public, colleagues in the workplaces, healthcare providers, and even PLHIV themselves. The findings further reveal the dual burden of stigma experienced by vulnerable populations such as children, adolescents, pregnant women, and key populations. Additionally, this review notes the presence of stigma marking directed at PLHIV, portraying them as dangerous, unclean, immoral, bad and irresponsible, and even sinners. In conclusion, in Indonesia, HIV stigma may take many different forms and occur in many different societal levels. This underscores the need for comprehensive, collective action and cross-sectoral interventions to effectively address these issues.

## 1. Introduction

Indonesia continues to face a significant public health challenge from HIV, with a cumulative total of 377,650 reported cases as of March 2023. Based on the type of population, 27.7% of new HIV cases identified in the country between January and March 2023 were among men who have sex with men (MSM), 3.3% were sex workers, 1.1% were transgender individuals, 0.5% were people who inject drugs (PWID), and the remainder were distributed across other populations such as pregnant women, individuals with high-risk partners, and others [1].

The life expectancy of people living with HIV (PLHIV) has significantly improved globally due to the widespread availability of antiretroviral therapy (ARV) [2,3], with life spans approaching those of the general population [4]. However, in Indonesia, challenges remain evident, including a low rate of viral suppression (reaching only 27%) [1] and high mortality rates among specific groups, such as an annual death rate of 10% among PLHIV in Bali [5]. These findings suggest that treatment success is influenced not only by ARV availability, but also by the effectiveness of the healthcare delivery system.

Indonesia has implemented various strategies to address the HIV epidemic in the country, including expanding the coverage of self-HIV screening, virtual interventions to reach key populations, access to Pre-Exposure Prophylaxis (PrEP) services, differentiated care services, “test and treat” strategies, and mentoring programs for healthcare professionals at health facilities [6]. Despite these efforts, the outcomes of these programs appear to fall short of the expected targets. As of March 2023, only 85% of PLHIV were aware of their status, 42% received antiretroviral therapy, and just 27% had achieved viral load suppression, indicating significant gaps in program effectiveness [1]. Evidence suggests that stigma and discrimination faced by PLHIV may have contributed to these challenges [7,8,9,10,11].

Stigma, as defined by Goffman, is a deeply discrediting attribute that alters an individual’s social identity, transforming them from being perceived as whole to being viewed as flawed and marginalized [12]. In the context of HIV, stigma involves negative attitudes and beliefs towards PLHIV [13]. The Joint United Nations Programme on HIV/AIDS (UNAIDS) broadens the definition of HIV-related stigma to encompass all forms of stigma and discrimination that affects the response to HIV, including factors such as gender identity, sexual orientation, drug use, sex work, and HIV status [14]. The HIV-related stigma poses significant barriers to the success of HIV prevention and control efforts. It has a profoundly negative impact on PLHIV, contributing to increased HIV prevalence [15], challenges in treatment adherence [16], decisions around HIV testing [17], and difficulties in disclosing HIV status [18]. At least two studies in Indonesia further confirmed this effect. A study in Papua showed that PLHIV who experienced stigma were less likely to adhere to their treatment (OR = 0.53; 95% CI = 0.32–0.89) [19]. Beyond its direct effect on HIV prevention programs, a study in Malang also found that PLHIV who reported lower levels of HIV-related stigma tended to have a better quality of life [20]. This indicates the needs to prioritize the development of interventions to address HIV-related stigma in Indonesia to support the country in achieving its targets in HIV mitigation programs.

The Health Stigma and Discrimination Framework, developed by Stangl et al. in 2019, emphasizes the importance of thoroughly understanding the manifestations and markings of HIV stigma to develop more specific and effective interventions. According to the framework, marking stigma refers to the process by which an individual is identified as having certain differences compared to others. This process is influenced by various factors, including lack of knowledge, socioeconomic status, prevailing norms or regulations, and other contextual elements. Typically, marking stigma is associated with aspects such as race, health conditions, gender, sexual orientation, occupation, and other social characteristics. The identification of these differences then triggers the application of stigma to the individual, which is referred to as stigma manifestation. Stigma manifestation encompasses the various tangible forms of stigma experienced, including denial of access to housing, verbal abuse or gossip, self-stigma, negative stereotyping, exclusion from social activities, and other discriminatory attitudes [21].

Stigma can stem from various sources, such as healthcare workers, family members, society, and even individuals PLHIV themselves. Therefore, understanding the patterns of stigma across different setting groups is crucial. However, to date, there has been no comprehensive review exploring the variations in HIV stigma manifestations and markings in Indonesia, including the patterns of stigma found in different settings. To fill this gap, this scoping review was conducted to systematically map and analyze these variations, providing a clearer understanding of how HIV stigma is expressed in Indonesia, as well as the stigma patterns that emerge across various settings.

## 2. Materials and Methods

This research is part of a broader review aimed at exploring HIV stigma in Indonesia, with the goal of providing a comprehensive overview of the manifestations and markings of HIV stigma in the country. This study adheres to the PRISMA for Scoping Reviews (PRISMA-ScR) guidelines [22] and is guided by the scoping review framework developed by Arksey and O’Malley [23]. The PRISMA-ScR checklist is available in the Supplementary Materials. While a review protocol was developed, it was not registered.

### 2.1. Literature Search

To identify relevant documents, a literature search was conducted from January to March 2024 using electronic databases covering studies from 2019 to 2023, along with other sources. The search strategy is presented in the following section.

#### 2.1.1. Electronic Databases

The search was conducted in PubMed, Scopus, and Google Scholar using combinations of various keywords synthesized from previous similar studies [15,24,25,26]. The list of keywords used can be found in the Supplementary Materials. In addition to using keywords, the literature search applied a publication date filter (2019–2023). To download the combined results of the literature search from Google Scholar and Scopus in CSV format, the researchers used the assistance of the Publish or Perish application [27]. The results of the literature from the search at PubMed were downloaded directly from the PubMed website.

#### 2.1.2. Reference List Searches

The reference lists of studies selected and passed the full-article screening stage were also examined to identify the additional literature.

### 2.2. Inclusion Criteria

Studies were included if they met the following criteria:

1. Published or conducted between 1 January 2019 and 31 December 2023: This criterion was applied to ensure that the data were relevant to the current situation of HIV stigma.
2. Written in English: This was performed to enhance the accessibility of the literature for global academic analysis.
3. Conducted in Indonesia: This criterion was used to obtain data specific to the context of HIV stigma in Indonesia.
4. Contained findings related to the manifestations or forms of HIV stigma in Indonesia, such as the type of stigma that emerged in specific contexts, the level or intensity of stigma in particular settings, and certain characteristics of PLHIV and key populations that were perceived as different by those enacting the stigma, thereby triggering its occurrence. This criterion was used to ensure that the reviewed literature was directly relevant to the main objectives of the review.
5. Not review research: Only primary research studies and those using secondary data sources were included, excluding the literature review.

In assessing the quality of the journals used, the researchers began by ensuring that the articles were published in internationally reputable journals. These journals were selected based on their high reputation and credibility, as well as the rigorous peer review process conducted by experts in the field, reflecting high editorial standards. Additionally, the researchers evaluated whether the research methodology was clearly and transparently explained and whether the research findings were relevant and aligned with the objectives of this scoping review. In this way, the critical appraisal process aimed to ensure that only studies of adequate quality and relevance were included in this review.

### 2.3. Literature Selection

#### 2.3.1. Abstract and Title Screening

All articles were imported into Microsoft Excel for processing. During the title and abstract screening stage, the author Ni Kadek Sudastri identified the presence of the keyword’s “stigma” and “HIV” within the titles and abstracts. This identification was facilitated using the “Search” function in Microsoft Excel. The keywords “stigma” and “HIV” were pre-determined based on a synthesis of keywords and terms commonly used in global studies on HIV stigma. Duplicate entries were identified at this stage through the “Conditional Formatting” feature in Microsoft Excel, which highlighted duplication based on similarities in titles and authors. The list of keywords used in the abstract and title screening can be found in the Supplementary Materials.

#### 2.3.2. Full-Article Screening

During the full-article screening stage, Ni Kadek Sudastri conducted a review of all the literature that passed the title and abstract screening. Ni Kadek Sudastri assessed the alignment of the literature with the predetermined inclusion criteria. In addition, a manual duplication screening was performed, during which Ni Kadek Sudastri examined similarities in titles, authors, and the overall content. The entire screening process was carried out using Microsoft Excel, which included an inclusion criteria checklist. Articles that met all the inclusion criteria at this stage were deemed eligible for inclusion in the review. The entire screening file was shared via OneDrive with all team members for review. To address the uncertainties that arose during the screening stage, the researchers engaged in discussions to reach a consensus in the decision-making process. Additionally, the screening process was carried out in stages to ensure that each piece of the literature considered met all the criteria established by the authors.

### 2.4. Data Charting

Data charting was performed using standardized forms approved by all research team members. The team members determined that the information charted was essential and contributed to the overall research findings. The charting process was conducted using Microsoft Excel, which was shared via OneDrive, allowing for all team members to review the charting process performed by each member.

In this study, the researchers included studies employing both quantitative and qualitative methods. During the identification stage, only quantitative studies that measured the percentage or score of HIV stigma within a population were included under the theme of the HIV stigma in Indonesia. For the theme of stigma manifestation, only quantitative studies that reported the percentage of individuals within a population engaging in behaviors classified as forms of HIV-related stigma were included in the analysis. These actions were subsequently grouped into more specific sub-themes within the broader category of stigma manifestation.

Meanwhile, for the qualitative literature, the researchers analyzed the phenomena presented in each study identified statements related to actions constituting HIV stigma or the differentiation of identity that led to the emergence of stigma. The findings from the qualitative literature were then organized into the themes of stigma manifestation and marking (identity differentiation). Findings from quantitative and qualitative studies that fell into the same category of stigma manifestation were grouped under a single theme. To achieve a more systematic presentation of the findings and to facilitate clearer information delivery, the researchers also developed sub-themes within each main theme.

### 2.5. Data Items

In this review, the information was categorized into three themes: manifestations, marking, and levels of HIV stigma. The manifestation of HIV stigma is the embodiment of HIV stigma marking, which can include experiences of stigma and discrimination, internalized stigma, perceived stigma, anticipated stigma, secondary stigma, stereotypes, negative prejudice, stigmatizing attitudes, and discriminatory behaviors. Conversely, marking refers to the process of identifying differences in an individual due to certain health conditions or perceived differences, such as race, social class, gender, sexual orientation, or occupation [21]. The manifestation of HIV stigma in this review was defined as any form of negative reactions or actions related to HIV exhibited by an individual or group towards PLHIV and populations associated with PLHIV. On the other hand, marking of HIV stigma refers to differences in individual characteristics directed at PLHIV, key populations, or their families, which then serve as the basis for the application of stigma. These differences may include factors such as race, gender, health conditions, sexual orientation, and other characteristics. Finally, the level of stigma was defined as the results of a quantitative study that indicates the prevalence or score of HIV-related stigma or discrimination in a particular region or setting.

### 2.6. Synthesis Result Method

In synthesizing the results, we used a thematic analysis approach [28], where the identified findings were grouped into relevant themes and then elaborated into a cohesive narrative. The process of forming group themes began with coding the review findings, categorizing the coding results and sub-themes, and establishing the main themes based on the identified categories and sub-themes. In grouping the themes, we followed The Health Stigma and Discrimination Framework to categorize findings into relevant themes or sub-themes.

## 3. Results

**Figure 1:**
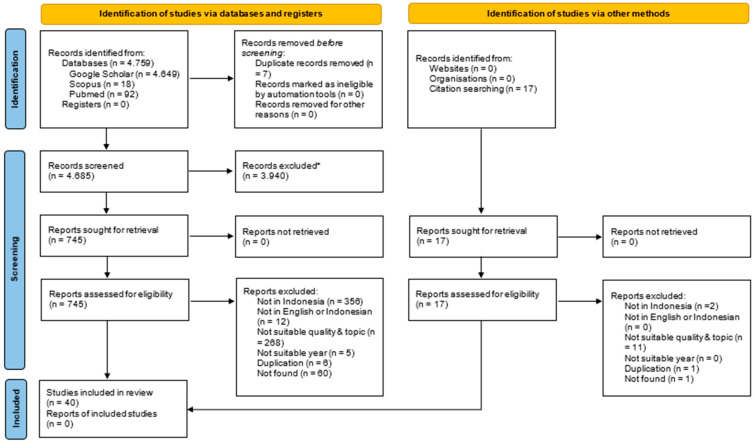
PRISMA flow diagram of the literature selection. * The literature was excluded because it did not contain the predefined keywords identified during the abstract screening process.

A total of 4776 articles were identified. Out of these, 745 passed the title and abstract screening stage, while 40 advanced to full-text article screening stage, resulting in 40 articles included in this review. Figure 1: PRISMA flow diagram of the literature selection providing more detailed information about the literature selection process.

Of the 40 studies included, almost a third (32%) were published in 2022. The majority of studies employed qualitative methods (63%), with data sources predominantly consisting of primary data (85%). Geographically, almost half of the research was conducted within the top 10 provinces with the highest number of PLHIV receiving ARV in 2021 (40%). Specifically, among these 10 provinces, the identified literature was conducted in eight provinces, which together contributed to 67% of PLHIV on ARV in Indonesia. Notably, a significant number of studies were conducted in West Java (12%) and Jakarta (12%). Despite contributing 12% to the total number of PLHIV on ARV in Indonesia and being among the top 10 provinces with the highest number of PLHIV on ARV, no studies were identified in Riau and Central Java, highlighting the uneven geographical distribution of research. More detailed information can be found in Table 1: Literature characteristics. All of the literature included in this review can be found in the Supplementary Materials.

The results were then divided into three main themes, including the following: the situation of HIV-related stigma in Indonesia, the manifestations of HIV-related stigma in Indonesia, and the markings of HIV-related stigma in Indonesia. The mapping of the types of HIV stigma manifestations and markings across various identified settings is presented in Table 2.

### 3.1. The Situation of HIV-Related Stigma in Indonesia

Studies indicated that HIV stigma was prevalent among family, healthcare workers, and the wider community, as well as among younger groups such as health-related students and youth. All of the literature identified under this theme consisted of studies employing quantitative methods. No studies were identified that measured the extent of HIV stigma issues quantitatively in other populations. Nevertheless, there is an imbalance in the number of studies measuring the magnitude of HIV-related stigma across the respective setting groups. Only one study specifically examined familial discrimination related to HIV [29]. In contrast, four studies were identified in the general population [30,31,32,33], two among youth [31,34], three among health-related university students [35,36,37], two among healthcare workers [35,38], and four among PLHIV or key population [7,39,40,41]. Interestingly, all studies measuring levels of familial discrimination, HIV stigma in the general population, and HIV stigma among youth utilized the same data source, namely the 2017 Indonesia Demographic and Health Survey (IDHS). Although some groups were represented in more than one study, the instruments or indicators used to measure stigma varied, limiting the ability to make direct comparisons across studies. Nonetheless, the identified literature consistently demonstrated that HIV stigma remained prevalent in all studied populations.

Familial discrimination was measured in one study that used data from the 2017 IDHS as its data source. The study assessed the extent of HIV stigma within families by analyzing respondents’ answers to questions regarding their willingness to hide a family member’s HIV status and feelings of shame associated with having a family member living with HIV. These responses were then defined as discriminatory behaviors related to HIV within the family. The study’s findings revealed a high prevalence of discriminatory behaviors related to HIV within families. Specifically, more than 70% of respondents from the general population exhibited such discriminatory behaviors [29]. This high proportion indicated that stigmatizing attitudes were not only limited to interactions with outsiders, but were also present within close family relationships. These findings suggested that the family, which ideally serves as a source of support, may have instead become a setting where stigma occurred, potentially exacerbating the psychosocial burden experienced by people living with HIV.

Furthermore, the measurement of HIV-related stigma at the general population level was identified in four studies. All four studies also utilized secondary data from the 2017 IDHS. Despite relying on the same data source, these studies employed different perspectives, focused on varying sociodemographic characteristics and used distinct HIV stigma indicators. Nevertheless, the findings across all four studies were consistent, revealing that more than 50% of study subjects exhibited discriminatory behaviors related to HIV. More specifically, over 60% of the total respondents demonstrated such behavior [30,32], with more than 50% of both male [31] and female [31,33] respondents engaged in discriminatory actions when analyzed by gender. This high prevalence of discriminatory behavior, across various sociodemographic groups and indicators, indicated that HIV stigma remained pervasive in the general population. Despite differences in study focus and methodology, the widespread nature of stigma was evident.

In addition to assessing the overall extent of HIV-related stigma in the general population, two studies found that HIV-related stigma behaviors were also present among young individuals. Using the same data previously mentioned, one study revealed that women aged 15–19 represented the highest proportion of those expressing discriminatory attitudes toward HIV compared to women in other age groups, 13.6% [31]. Additionally, another study with the same data source found that 85.9% of respondents aged 15–22 demonstrated stigmatizing attitudes [34]. Although using the same data source, these studies revealed significant differences in the prevalence of HIV stigma among youth. A lower prevalence of stigma was observed in women aged 15–19 years compared to the higher percentage found in the broader 15–22 age group. This variation may be associated with age and gender factors, which could influence the expression of stigma within this demographic group.

In addition to measuring the percentage of respondents using HIV stigma indicators, several studies assessed the extent of HIV-related stigma through scoring systems. One commonly employed scoring method is The AIDS-Related Stigma Scale [42]. This instrument consists of 12 self-reported questions designed to evaluate public perceptions of HIV-related stigma toward individuals living with HIV. Responses were recorded using a four-point Likert scale ranging from “strongly disagree” to “strongly agree”. The total score ranges from 12 to 48, with higher scores indicating higher levels of stigma. Utilizing this method, one study reported that the average HIV stigma score among pharmaceutical students from Java was 21.02 ± 4.65 [35]. This indicated that the stigma scores did not exceed half of the maximum HIV stigma score, suggesting that the HIV stigma levels in these groups were relatively low.

Another scoring method used to measure the extent of HIV-related stigma was through a questionnaire developed by researchers based on prior preliminary studies. Using this method, one study developed a questionnaire consisting of 16 items, evaluating using a Likert scale ranging from 1 (strongly disagree) to 5 (strongly agree). The maximum total score that could be achieved was 80. The cutoff value to identify supportive attitudes was set at a score of 20, where higher scores indicate less supportive or more stigmatizing attitudes. This study found that 99.6% of dietetic students from three universities on Java Island reported high stigma scores, with a mean score of 42.7 ± 0.27 [36].

Scoring methods were also employed to measure the extent of HIV-related stigma among nursing, midwifery, and medical students in Jakarta, Surabaya, and Jayapura. In this study, HIV stigma was assessed using the Nurses’ Attitudes AIDS Scale (NAAS). This instrument consisted of 41 questionnaire items covering attitudes toward homosexuality, attitudes toward people who use drugs, willingness to provide care for PLHIV, and beliefs about workplace and societal restrictions for PLHIV. Each item was rated on a five-point Likert scale, ranging from strongly agreed to strongly disagreement. The HIV stigma score was obtained by calculating the average score of participants across each of the 41 items. This study found that nursing, midwifery, and medical students had an average stigma score of 3.15, with 5 being the maximum possible score [37]. This indicated that the level of HIV stigma found across all student groups falls within the moderate to high category.

The findings indicate that HIV stigma levels varied across health-related student groups. Pharmacy students showed low levels of stigma, while dietetics students demonstrated high stigmatizing attitudes. Meanwhile, nursing, midwifery, and medical students generally fell within the moderate to high stigma category. These differences were likely influenced by the specific disciplinary contexts and the measurement tools used in each study.

Two studies were identified that specifically measured the extent of HIV stigma within healthcare facilities. The first study, conducted in Gunung Kidul and focused on healthcare workers in community health centers and a hospital, assessed HIV stigma through four key indicators: expressing fear of contracting HIV from PLHIV, holding negative perceptions toward PLHIV, refusing to provide healthcare services to PLHIV, and engaging in discriminatory practices by taking excessive precautionary measures when treating PLHIV. This study found that more than 50% of healthcare workers in both community health centers and hospitals expressed fear of contracting HIV from PLHIV, held negative perceptions toward PLHIV, refused to provide healthcare services to them, or engaged in discriminatory practices by taking excessive precautions while treating PLHIV [38]. The second study employed the AIDS-Related Stigma Scale to measure the level of HIV-related stigma among pharmacy staff at 250 healthcare facilities across 33 provinces in Indonesia. The results show that the median HIV stigma score among pharmacy staff was 20.66 ± 4.41, which fell into the low category [35].

The findings from both studies indicate the presence of HIV-related stigma among healthcare workers, although the extent varied across settings and measurement approaches. In the first study, more than half of the healthcare workers in both community health centers and hospitals demonstrated stigmatizing attitudes or behaviors across multiple indicators. This suggested a widespread manifestation of stigma among different types of health professionals. Meanwhile, the second study reported a relatively low median stigma score (20.66 ± 4.41) among pharmacy staff, based on a standardized stigma scale. The contrast in results may reflect differences in the nature of the healthcare roles, institutional contexts, or stigma measurement tools applied in each study. These findings underscore the complexity of assessing stigma within healthcare settings and the need to consider various contextual and methodological factors when interpreting stigma levels.

The measurement of HIV-related stigma found in the studies not only examined the perspective of those who perpetuated the stigma, but also considered the extent of stigma reported or experienced by those who received it. The studies involved a diverse range of respondent groups, including MSM, women living with HIV (WLHIV), and PLHIV in general. Two studies found a relatively high percentage of MSM experiencing HIV stigma. Specifically, 50% of MSM in two healthcare facilities located in Jakarta reported high levels of HIV-related stigma [39]. Another study in Bukittinggi City found that 82% of MSM reported experiencing HIV-related stigma and discrimination [40]. Stigma was not only prevalent among MSM, but studies also found that HIV-related stigma was notably high among women living with HIV. Approximately 50.8% of women with HIV at Dr. H. Abdul Moeloek General Hospital reported high levels of HIV-related stigma [7]. However, although three studies found relatively high levels of HIV stigma in certain groups across various locations, the extent of HIV stigma among PLHIV in general was found to be low in a study conducted in Bali, with a mean score of 42.88 (SD ± 17.59), while the maximum possible mean score was 100 [41].

The findings indicate varying levels of HIV-related stigma, with higher levels consistently observed among vulnerable groups such as MSM and women living with HIV. Both studies focusing on MSM reported consistently high levels of experienced stigma. Similarly, more than half of women living with HIV in one study reported experiencing high stigma. In contrast, a study involving the general PLHIV population in Bali reported a relatively low average stigma score. This variation may reflect the influence of group vulnerability and geographic-cultural context on the experience of stigma.

### 3.2. The Manifestations of HIV-Related Stigma in Indonesia

A range of variations in the manifestations of HIV-related stigma was noted in some studies. The elaboration of findings on this theme will be divided into six sub-themes, as follows.

#### 3.2.1. Avoiding Contact with PLHIV as a Common Manifestation of HIV-Related Stigma

Although the manifestations of HIV stigma vary depending on the location, avoiding contact with PLHIV might be the most common form of HIV stigma. Six studies, all employing a qualitative approach, found that the act of avoiding interaction with PLHIV occurred at the family level. The fear of contracting HIV often led members to physically distance themselves from PLHIV [43,44,45]. Family members had also explicitly prohibited PLHIV from approaching or making contact with their children [45,46], or even refused to visit PLHIV [47]. In addition to the avoidance of direct physical contact with PLHIV, another form of avoidance found within families was the reluctance to handle items associated with PLHIV. Some family members refused to touch the clothing of PLHIV due to the mistaken belief that HIV can be spread this way [48]. Spouses of PLHIV also shared instances where family members moved out of their nearby homes, fearing that HIV could be transmitted through shared water sources [47].

Beyond the family setting, nine studies, all employing a qualitative approach, found that the avoidance of physical contact with PLHIV was also practiced by the community members surrounding PLHIV. Instances of physical avoidance have been reported by PLHIV, particularly by neighbors, who refrained from shaking hands or sitting in the same chair [10,43,48,49,50], touching their hands, eating together [10], or even communicating with them [51]. There were PLHIV who shared their experience of attending a public place of worship where no one was willing to shake hands with them, despite handshaking being common among others [49]. Furthermore, society often viewed PLHIV as violators of social norms, to the point that people avoided even walking past their homes [50]. Furthermore, regarding the avoidance of contact in the community, two studies reported that communities also refused to consume food touched by PLHIV [46,49]. This avoidance behavior extended even after PLHIV’s death. This was expressed by healthcare workers in a qualitative study, stating that when a PLHIV passed away, the entire community refused to consume the food and drink served, avoided approaching the body, and wore masks out of fear of transmission [43]. This widespread avoidance has led many PLHIV to hesitate in disclosing their HIV-positive status, fearing social ostracization [44]. Along with misconceptions about how HIV is transmitted, a study also highlighted a community’s limited understanding of ARV treatment. Despite adhering to ARV treatment, PLHIV reported that community members continued to avoid contact with them out of fear of HIV transmission [9].

Regrettably, two studies that used qualitative methods found that some instances of contact avoidance escalated to the outright expulsion of PLHIV. Experiences shared by PLHIV revealed that disclosing their HIV status to the family provoked anger within the family, leading to near-expulsion [49] not only by family members, but also from their villages by the community once their HIV status became known [8].

Avoidance of contact by healthcare workers was also reported, as admitted by the healthcare workers, peer support groups, and PLHIV themselves. Eight studies were identified that indicated contact avoidance actions carried out by healthcare workers. All of these studies employed a qualitative approach. Three studies noted that healthcare workers acknowledged that many of their colleagues in healthcare facilities hesitated and refused to serve PLHIV [52], often citing reasons such as lack of knowledge and fear of contracting HIV, leading them to avoid physical examinations [43,53]. In another study, peers supporters also reported instances where some healthcare workers refused to administer treatments involving the genital areas of PLHIV, such as inserting medication [54]. Denial of services by healthcare workers upon learning someone’s HIV status was also reported by PLHIV [55].

Beyond direct refusals, PLHIV often faced indefinite delays in receiving non-HIV-related healthcare services once their HIV status became known [49]. Another study in Papua found that healthcare workers’ refusals to provide care provoked anger among PLHIV, who demanded their right to access services, leading to a doctor’s eventual intervention [11].

The avoidance of PLHIV in healthcare settings was found not only to be experienced by patients, but also by PLHIV working in healthcare services. A study noted that a PLHIV’s wife recounted how her husband was shunned by his colleagues despite working at a primary healthcare facility, demonstrating how HIV stigma can also affect PLHIV in the workplace [47].

Based on the findings presented above, contact avoidance as a form of HIV stigma was identified among various groups, including family members, community members, and healthcare workers. A similar pattern of avoidance was observed across all populations, involving the avoidance of physical contact with the body of PLHIV, as well as with objects that had been touched by PLHIV. The various forms of avoidance observed were likely caused by a lack of understanding regarding the actual modes of HIV transmission. These misunderstandings were often linked to fears of transmission. As a result, PLHIV frequently experienced discriminatory treatment in the form of limited physical interaction and social exclusion from those around them.

#### 3.2.2. Different Treatment by Those Willing to Interact with PLHIV

Studies indicate that not all individuals who stigmatized PLHIV completely avoided contact with them. However, those who did maintain contact often subjected PLHIV to different, and at times discriminatory, treatment. This behavior was observed both amongst family and in healthcare providers. A total of four qualitative studies described how differences in the treatment of PLHIV emerged within families. In two studies, PLHIV reported that, even while living with their families, they experienced differential treatment from other family members. This included the separation of personal items, such as dining utensils, which were specially marked for them [43,49]. The practice extended to food and water designated for PLHIV, with one study stating that with some family members required PLHIV to wash their dishes and clean the toilet after use [49]. There were even instances where food was delivered to PLHIV through a gap under the door, as though feeding a confined pet [43,49]. Some families even boiled PLHIV’s clothes before washing them to prevent transmission to other family members [49]. Feelings of isolation and abandonment were expressed in response to the separation of personal items by family members, as shared by PLHIV [43]. Ironically, this separation of personal items occurred even in families that otherwise supported the PLHIV’s treatment [11]. To overcome stigma, PLHIV often chose to isolate themselves, driven by the harsh treatment they faced from their families after disclosing their HIV status [56].

In healthcare settings, PLHIV often experienced differential treatments, particularly through excessive self-protection measures taken by healthcare workers. Three qualitative studies described the occurrence of differential treatment toward PLHIV in healthcare settings. For example, some healthcare workers were reported to wear double gloves and repeatedly wash their hands after providing care, making PLHIV feel stigmatized and dehumanized [48]. Furthermore, PLHIV reported being placed on beds at the end of the room, separated from other patients. Some also recounted being rushed to complete their treatment even when their condition was not stable, leading to their re-admission to the hospital due to deteriorating health. In certain cases, these individuals were then placed in isolation rooms under the justification of having a dangerous HIV-related disease [55]. The same was found in another study, with PLHIV only being allowed hospitalization if they agreed to be placed in a private room [52].

In addition to direct discrimination from individuals within the home setting and healthcare facilities, one qualitative study found that PLHIV also experienced differential treatment in the workplace. This included being dismissed or rejected from employment at certain institutions due to HIV status, leading PLHIV to prefer working in NGOs related to their health condition or starting their own business to avoid HIV-related stigma in traditional work environments [52].

Differential treatment as a manifestation of HIV-related stigma was identified across various groups, including family members, healthcare workers, and workplace institutions. Nevertheless, this form of stigmatization could be considered less severe than complete contact avoidance, as there remained a willingness to interact or maintain connections with PLHIV, albeit in a manner different from interactions with others. Such differential treatment was generally manifested through the segregation or restriction of the rights of PLHIV, aimed at minimizing direct contact. When viewed in light of the forms of treatment identified, it is highly likely that this behavior was influenced by fear and a lack of proper understanding regarding the actual mechanisms of HIV transmission.

#### 3.2.3. Negative Social Reactions to PLHIV

Another form of HIV stigma manifestation identified in the study was negative social reactions. Four qualitative studies described various forms of negative social reactions exhibited by the community. Negative social reactions to PLHIV were sometimes manifested through unsympathetic comments from community members, often their closest friends. These individuals, aware of the PLHIV’s past risky behaviors, remarked that the HIV status of both the PLHIV and their daughter was a punishment for their past actions, specifically having multiple sexual partners before marriage. These comments left the PLHIV feeling shocked and depressed, as they had not expected such negative views from those closest to them [57]. Additionally, gossip about PLHIV’s status was also prevalent within the community. Individuals living with HIV shared that, for years, their HIV status had been the subject of gossip [49,50]. This gossip was not confined to individuals without a healthcare background, but was also spread by healthcare professionals, who disclosed the PLHIV’s HIV status to the community, contributing to the spread of information within the community [8,43,49].

Altogether, four qualitative studies identified various forms of negative social responses displayed by healthcare workers. In addition to gossiping, three studies observed another adverse social reaction in healthcare settings. Healthcare workers often expressed anger towards PLHIV while providing care. Some healthcare workers reported feeling particularly frustrated towards PLHIV who contracted HIV through risky sexual behaviors, such as engaging with commercial sex workers, despite having a legitimate partner. This anger was driven by sympathy for the PLHIV’s partners, who had to endure the consequences of their spouse’s infidelity and the associated emotional pain [43]. Furthermore, in one study, PLHIV recounted instances where healthcare workers displayed anger and physical violence upon learning of their HIV status after receiving healthcare services. In that study, a PLHIV noted that healthcare workers expressed a preference for treating Coronavirus Disease 2019 (COVID-19) patients over HIV patients, even during the COVID-19 pandemic [58]. Additionally, some PLHIV reported being reprimanded by a midwife during their pregnancy, who asserted that a PLHIV should not be pregnant, leading to emotional distress for the PLHIV involved [47].

Healthcare workers in healthcare settings also often make unsympathetic comments towards PLHIV and key populations. These callous comments included questions or statements that offended PLHIV. For instance, two studies found that some healthcare workers admitted that their colleagues intentionally asked probing questions related to the risky behaviors that led to the PLHIV contracting HIV, such as “Why are you gay?” [49] or accusing the PLHIV’s husband of having had sex with a sex worker [47].

Negative social reactions towards PLHIV were identified not only among the general public, but also within professional environments that should have provided support and protection. These reactions were often triggered by a lack of understanding about HIV. Unlike the forms of HIV stigma previously identified, negative reactions towards PLHIV were likely also linked to certain beliefs, including religious teachings and social norms held by the individuals involved. These beliefs exacerbated perceptions and treatment of PLHIV, which in turn reinforced stigma and triggered negative reactions towards them.

#### 3.2.4. Self-Stigma

There were seven qualitative studies that described the self-stigma experienced by PLHIV. The review findings reveal that experiences of stigma encountered by PLHIV evoked significant fear and anxiety. This condition drove them to avoid locations perceived as potential sources of stigma, including healthcare facilities, which in turn might have hindered their access to essential care and treatment. Healthcare providers reported that one of the reasons for PLHIV’s non-compliance with ARVs was their fear of the challenges they may face when accessing services [9]. Concerns about being ostracized after disclosing their condition, including fears that healthcare providers might refuse to offer care, were also expressed by PLHIV [9,58].

Not limited to healthcare services, the review findings also indicate that PLHIV often experienced significant fear and anxiety when disclosing their HIV status to family members. Individuals living with HIV feared negative reactions from their family members upon disclosure of their status, including discrimination similar to that experienced by others, such as being rejected by their family and community during burial rituals [48]. This rejection not only led to the avoidance of interactions, but also created reluctance among PLHIV to disclose their status due to the fear of being subjected to various forms of bullying from their friends [59].

In addition to fear and anxiety, studies also identified self-stigma in the form of a loss of self-confidence. One study highlighted that PLHIV reported losing pride in themselves and feeling low self-worth [51]. This loss of pride was expressed by PLHIV because of missed opportunities to achieve personal aspirations due to their HIV-positive status, which ultimately led to feelings of sadness and trauma. Some PLHIV also disclosed that HIV caused so much suffering that they even considered ending their lives to escape it [60].

In addition to the feelings mentioned above, studies revealed that many PLHIV experienced feelings of guilt and sinfulness, especially those who engaged in risky sexual behavior. These feelings were intensified by societal stigma and discrimination, particularly in a culture where PLHIV were perceived as unclean, and associated with other stigmatized communities, such as MSM, transgender, sex workers. As a result, PLHIV felt that they were being judged as part of these communities, regardless of their identity [48]. One study noted various ranges of emotions upon learning their HIV status. Some PLHIV could accept their diagnosis due to the acknowledgment of their past behaviors. Nevertheless, they remained concerned about the potential impact on their spouse and children, who may also be infected [61].

Findings from studies on self-stigma among PLHIV indicated that HIV-related stigma not only hindered their access to healthcare services, but also had a profound impact on their mental and emotional health. External and internal stigma created a cycle of fear, avoidance, despair, and guilt, which ultimately deteriorated the quality of life of PLHIV.

#### 3.2.5. HIV Stigma Among Children, Adolescents, Pregnant Women, and Key Populations

HIV stigma can significantly harm children, adolescents, pregnant women, and key populations. These groups were specifically highlighted because they had already been categorized as vulnerable populations even before being affected by HIV, which further heightened their susceptibility to stigma and its negative impacts.

Among children living with HIV (CLHIV), stigma hindered their access to healthcare services. There are five qualitative studies that show how stigma related to HIV might impede CLHIV in several ways. One study found that healthcare providers refused to offer services to CLHIV, demanding someone with HIV-negative status to accompany the child for ARV services [46]. Additionally, another study noted that healthcare providers were reported to have used double gloves when providing services to children because their parents were PLHIV [49]. In another instance, PLHIV recounted their experience of their child, also living with HIV, being denied access to the hospital restroom during a healthcare visit [49,57]. Similarly, one study noted an occurrence where children of PLHIV were placed in isolation, such as being assigned to the end bed in wards reserved for B20 patients, with healthcare providers responsible for changing bed linens donning full personal protective equipment including gowns, masks, and double gloves [55]. These examples highlight the significant challenges faced by children of PLHIV in accessing healthcare services due to their or their parents’ HIV status.

Not only did they face barriers due to HIV-related stigma from healthcare workers, but CLHIV who became aware of their HIV status often experienced feelings of disappointment, shock, and sadness. Some families, in an attempt to shield their children from stigma, even restricted them from forming friendships to prevent their HIV status from being disclosed. Another effort made by families to conceal their child’s HIV status, included avoiding access to government-provided services, such as formula milk assistance, health insurance, and peer support groups, further isolating the children from potential sources of support [62].

HIV-related stigma not only affected PLHIV, but also significantly impacted key populations, creating profound barriers to accessing healthcare services and support. This finding was identified in two quantitative studies and four qualitative studies. A study revealed that almost half (11 out of 29) of MSM experienced rejection from family members, adding to the challenges they faced in seeking support [63]. Within healthcare settings, stigma was equally pervasive. In Gunung Kidul, 64% of community health center staff admitted to refusing services to key populations whenever possible [38]. Additionally, 9 out of 209 MSM reported being rejected outright by healthcare providers, many of whom expressed discomfort or disgust during their first interactions with MSM patients [53]. Verbal stigma and moral judgments further exacerbated the issue. Three studies found that individuals who disclosed their sexual orientation to aid in diagnosis were often met with lengthy religious lectures, with their orientation described as sinful [9,43,64].

Although studies on HIV stigma among adolescents were limited, one quantitative study found that 11 out of 29 young people living with HIV experienced aversion or rejection from their family or friends [63]. This suggests that adolescents within key populations or those living with HIV may face similarly severe impacts from HIV stigma.

HIV-related stigma also affected pregnant women. While it was not commonly found among studies included in this review, one qualitative study reported that a pregnant woman, although HIV-negative herself, was denied healthcare services during her pregnancy check-ups due to her husband’s HIV-positive status [47]. This showed how a partner’s HIV status could affect a pregnant woman’s access to healthcare service. Additionally, pregnant women also faced significant human rights violations. One qualitative study recounted the experience of a 22-year-old woman living with HIV who was coerced into signing a sterilization consent form before delivering her child, with the threat that surgery would not proceed unless she complied [52]. This example illustrates how HIV can intersect with human rights.

The identified findings indicate that vulnerable populations experienced various forms of stigma, such as avoidance of contact, differential treatment, negative reactions, and the emergence of self-stigma. The impact of stigma became increasingly severe, as it not only hindered their access to healthcare services, but also obstructed the fulfillment of their fundamental rights as vulnerable groups. As previously mentioned, for instance, some parents were reluctant to enroll their children in health insurance programs or apply for formula milk assistance due to fears that their child’s HIV status might be disclosed. The various forms of stigma identified appeared to be associated with a lack of knowledge regarding HIV transmission, as well as influenced by prevailing social norms, cultural values, and religious beliefs.

#### 3.2.6. Gradual Changes in HIV-Related Stigma Within Communities

Two qualitative studies also revealed positive outcomes, indicating a change in how HIV stigma was perceived within society. Although not frequently reported in the literature, fortunately, improvements have been seen, with one study observing that, while people were once completely unwilling to interact with PLHIV, they were at least willing to engage in conversation with them, albeit from a certain distance [47]. Similarly, a study found that some community members claimed that they would behave kindly towards PLHIV, but still kept their distance [51]. These findings indicate that there has been progress in reducing HIV-related stigma; however, acceptance of PLHIV remains partial. Although the community has begun to show signs of openness, a persistent sense of caution suggests that stigma still exists, albeit in a more subtle form.

### 3.3. The Markings of HIV-Related Stigma in Indonesia

Stigma marking is the process of identifying an individual’s differences, which is influenced by driving and facilitating factors that ultimately shape the form of HIV stigma that occurs. Nine qualitative studies have successfully identified differences in the characteristics of PLHIV, which subsequently trigger the emergence of different forms of HIV stigma. Studies indicated that PLHIV were not only identified as individuals with HIV, but were also associated with negative characteristics related to the perceived modes of HIV transmission according to societal beliefs. Healthcare providers conveyed that families and communities perceived PLHIV as dangerous individuals, largely due to the perception of HIV as a severe disease [43,45,50,51]. Some families also believed that an individual’s HIV status was a form of divine curse for what they perceived as “dirty” behavior, including drug use, sex work, and other actions deemed sinful [49,62]. Additionally, PLHIV expressed that society’s understanding of HIV transmission was limited, associating it primarily with unprotected sex or sex work. Hence, PLHIV were often marked as immoral individuals, reinforcing the stigma tied to promiscuous behavior [49].

One study reported occurrences where PLHIV reported being labeled as “bad mothers” [8]. This misconception stemmed from the belief in society that “good” mothers cannot contract HIV, with HIV viewed as a “dirty” disease transmitted through promiscuous behavior. One of the studies included in the review also highlighted the experiences PLHIV during childbirth, where, upon disclosing their HIV status to healthcare providers, it led to immediate moral judgment from the healthcare providers. These women were further perceived as irresponsible mothers, primarily due to assumptions about their engagement in promiscuous behavior. However, PLHIV expressed that, by disclosing their status, they aimed to protect their children from HIV infection [58]. Similarly, three studies stated female PLHIV often being stigmatized as “hina” or morally degraded women [49,62]. On the other hand, a study found that the community viewed being HIV positive as being caused by risky sexual behavior and as punishment from God [62].

The HIV stigma marking found in the literature referred to the process of identifying PLHIV or key populations as individuals perceived as bad or dangerous. This was likely influenced by an individual’s understanding of HIV transmission methods, as well as their perception of the current HIV situation, and further reinforced by prevailing social norms and religious values. The impact is not limited to key populations, but also extends to the general public, who may not be involved in any risky behaviors.

## 4. Discussion

This study aims to explore the manifestations and markings of HIV-related stigma experienced by PLHIV, while also mapping its patterns across various settings in Indonesia. This review reveals that, while numerous studies on HIV stigma have been conducted in Indonesia, their geographic distribution remained uneven. For instance, in 2021, the Riau Islands and Central Java recorded 4678 and 19,944 PLHIV receiving ARV, respectively, out of a total of 204,542 PLHIV accessing ARV services in Indonesia [65]. This means that approximately 12% of the national PLHIV population accessing ARV services were concentrated in these two provinces. The number of PLHIV on ARV in a region may reflect both significant healthcare access among PLHIV and, indirectly, a substantial population of PLHIV. Nevertheless, research on HIV-related stigma in the Riau Islands and Central Java remains very limited.

Several studies have indicated that resource allocation plays an important role in determining research output in a given region [66,67,68]. Referring to these findings, the limited number of studies on HIV-related stigma in the Riau Islands and Central Java provinces in this study was likely attributed to the scarcity of resources. In this context, resources included limited research funding, the number and capacity of researchers interested in HIV stigma issues, restricted access to target populations, inadequate research infrastructure, and administrative and regulatory barriers at the local level.

Based on these findings, strategic efforts are needed to enhance research capacity in these regions. It is recommended that local governments establish closer partnerships with universities, research centers, or local research institutions to strengthen collaboration networks and expand the scope of research related to HIV stigma. Additionally, providing incentives for researchers, allocating dedicated research grants, and creating policies that facilitate data access and protect key populations are considered. The enhancement of research capacity in the targeted regions is expected to contribute to a better understanding of HIV-related stigma at the local level, thereby enabling the development of more tailored and effective stigma reduction strategies.

This review finds that HIV-related stigma occurs across various settings in Indonesia, including from family members [29], healthcare workers [35,38], the general public [30,31,32,33], youth [31,34], health-related students [35,36,37], and PLHIV [7,39,40,41]. Although several studies were conducted to examine stigma in these populations, there is still a lack of quantitative research measuring the extent of HIV stigma outside healthcare institutions, among students in non-health fields, and other populations that had not been extensively studied. These populations may also serve as environments that influence the stigma experiences of PLHIV. Additionally, an imbalance in research was found particularly regarding HIV stigma at the family level, which remained significantly underexplored. The use of similar secondary data, such as IDHS 2017, in studies on youth and the general public may have narrowed the scope of information, as the data came from similar populations. Therefore, measuring the extent of HIV stigma across various populations in Indonesia remains critically needed. To address this, stronger collaboration between government, academia, and the community is necessary to map populations that have not been reached by HIV stigma research. Furthermore, further studies should prioritize these populations. By using a more holistic approach that considers the perspectives of various populations, regions, and data variations, more effective and targeted interventions can be developed.

Reports of stigma related to HIV were widely reported across various population groups, including healthcare workers [35,38], communities [30,31,32,33], families [29], and others, with varying degrees of severity ranging from low to high [35,36,37]. From the perspective of PLHIV, more than 50% in each study conducted reported that they had experienced or were still experiencing stigma related to their HIV status [7,39,40]. These findings aligned with the Global HIV Stigma Index 2.0, which indicated that, globally, 13% of PLHIV still experienced stigma when accessing healthcare services, and this figure increased significantly in the context of community settings, reaching 23.6% [69]. Similar findings were reported in a study conducted in Myanmar, where 47% of PLHIV experienced enacted stigma and 87% reported internalized stigma [70]. In contrast, studies in the United States showed that HIV-related stigma was relatively rare among PLHIV when accessing healthcare services [71]. These differences suggested that the magnitude of HIV stigma may be strongly influenced by contextual factors such as culture, social norms, and the presence of regulations that protect the rights of PLHIV. In addition, variations in research methods was likely contributing reasons for the differing levels of HIV stigma identified across various settings in Indonesia.

From the perspective of manifestations, different patterns of HIV-related stigma emerged across various settings. In healthcare facilities, stigma was manifested through avoidance of contact, differential treatment, and negative reactions toward PLHIV and key populations. At the community level, stigma tended to take the form of avoidance and negative reactions, with differential treatment being less commonly observed. Within families, manifestations of stigma included both avoidance and differential treatment. Among PLHIV themselves, stigma was predominantly internalized, often expressed as self-stigma. Additionally, stigma was also found in other institutions, particularly regarding the restriction of PLHIV’s rights to access employment. These various manifestations of stigma can significantly undermine the ability of PLHIV to exercise their fundamental rights, especially among those facing intersecting vulnerabilities. Addressing such stigma across all levels and institutions is essential to ensuring equitable access to health, social, and economic opportunities for PLHIV.

Most of the causes of this stigma stem from a lack of knowledge or misunderstandings regarding HIV transmission [9,43,46,47,53]. This was also evident in the labeling of HIV stigma, where the process of distinguishing PLHIV and key populations led to their identification as ‘bad’ individuals. This judgment was rooted in associations with behaviors considered risky and seen as deviating from prevailing religious and social norms. Additionally, PLHIV were perceived as dangerous due to widespread misconceptions within communities about how HIV is transmitted.

Various educational methods have been identified in the literature and were considered to have a positive impact on reducing stigma and discrimination against PLHIV. One commonly used method is the dissemination of information through social media, which was regarded as effective in reaching both PLHIV and the general public, particularly those interested in the issue [9]. Additionally, educational efforts were carried out through collaborations between healthcare facilities and non-governmental organizations (NGOs), which engaged directly with communities to provide information on HIV-related services and basic HIV knowledge [49,64]. Personalized educational approaches, such as one-to-one counseling, were also implemented, where counselors provided direct education to PLHIV and their families. These sessions took place not only at healthcare facilities, but also during home visits, particularly when PLHIV reported experiencing stigma within their families [46,48].

The presence of dedicated facilitators for PLHIV in healthcare settings was also found to be beneficial in helping individuals and their families address stigma and service-related barriers. These facilitators offered tailored education, explained service procedures, and accompanied PLHIV during healthcare visits [54]. Moreover, PLHIV who participated in peer support groups or had peer companions often felt more motivated and had a safe space to discuss HIV-related challenges [11,46,60]. Being part of an organization was also perceived positively by some PLHIV, as it provided them with a sense of legitimacy and greater acceptance within the community [64]. Educational interventions were not limited to PLHIV, families, and communities, but also targeted healthcare providers through HIV training programs, which included basic knowledge and guidance on providing appropriate care to HIV patients [43].

However, despite the positive outcomes reported in various studies, only a limited number evaluated the effectiveness of these interventions using quantitative methods. Most findings remained qualitative in nature. Furthermore, the implementation of these interventions was uneven across Indonesia, resulting in limited access to similar programs in many regions. Another important gap identified was the lack of interventions addressing social and religious norms, even though these factors played a critical role in shaping stigma, as highlighted in this review. Therefore, there is a need to strengthen research capacity to evaluate intervention effectiveness more comprehensively, as well as to develop innovative and accessible models. For example, web-based training for healthcare providers could offer continuous education that is available anytime and anywhere.

Moreover, the educational content should focus on addressing the common misconceptions and misunderstandings that still prevail within the general public. The lack of understanding regarding HIV also leads to various negative labels being assigned to PLHIV, often based on misconceptions about HIV transmission [8,47,49,50,58,62]. HIV can be contracted through routes that should not be deemed immoral acts. For example, a child may acquire HIV from their parents, a wife from her spouse, or a healthcare worker who is accidentally infected through a needle-stick injury. This aspect should be emphasized in the educational content provided to the general public. In addition to understanding HIV transmission, it is equally important for the public to recognize the positive impact of ARV for PLHIV. ARV as prescribed can suppress HIV viral load in PLHIV, not only benefiting their health, but also reducing the risk of transmission to others [72]. This knowledge is essential for the general public to foster increased support for the treatment and well-being of PLHIV.

To ensure that educational interventions are accepted and align with community values, it is crucial to adopt an approach that resonates with local religious and social norms. In this context, involving respected religious leaders and traditional community figures in the educational process could be an effective strategy. Their participation would help bridge the gap between scientific knowledge about HIV and the cultural perspectives held by the community. By leveraging the influence of these community leaders, it becomes possible to create an environment where information about HIV transmission is more readily accepted and internalized, fostering a shift in attitudes toward PLHIV and key populations. This approach not only enhances the effectiveness of stigma-reduction programs, but also encourages broader community support, ensuring the sustainability of such interventions over time.

In addition to reflecting inadequate dissemination of information related to HIV, the forms of stigma found in this review suggest that PLHIV often faced limitations on their basic rights. For instance, within healthcare services, PLHIV encountered refusal or delays in receiving care, as well as the use of derogatory language in response to their HIV status. Such behavior highlights violations of patients’ rights to equitable and inclusive healthcare [73]. Moreover, this behavior of HIV-related stigma extended beyond adult PLHIV to other vulnerable groups such as CLHIV, Adolescents Living with HIV (ALHIV), pregnant women with HIV, and other key populations. Therefore, providing educational training on HIV stigma alone is insufficient. Training should also include communication, interaction, and care provision guidelines for PLHIV and vulnerable populations to ensure more inclusive services and better protection of patient rights. In addition, the presence of strong regulations to protect the human rights of PLHIV may also be necessary to ensure the equality of rights for PLHIV in healthcare settings, society, and the workplace.

This review indicates that the knowledge and perceptions of those who perpetrate stigma might be the key factors in why individuals engaged in stigmatizing actions or held stigmatizing attitudes. However, various other studies have identified additional factors contributing to the formation of HIV stigma, such as residential location [30,33,48] and workplace setting [38,74]. Given this, the intervention approaches developed should not solely focus on increasing knowledge and changing perceptions through one-time educational efforts. A more comprehensive approach, such as a collective action approach involving the participation of multiple sectors to achieve shared goals, could be an effective strategy to address HIV stigma in Indonesia. Collective action allows for individuals or groups affected by HIV to collaborate in overcoming the social, cultural, and systemic barriers that hinder access to healthcare services. Similarly to the study conducted in Thailand, the potential of collective action was demonstrated through efforts by the MSM community and male-to-female transgender individuals living with HIV. In this study, the community worked together to learn from each other and teach strategies for combating stigma, including stigma originating from social environments, families, and healthcare providers [75].

Studies indicated that PLHIV perceived certain healthcare procedures as conveying a sense of stigma towards them, such as placement in isolation rooms or separate areas, as well as the use of B20 labeling [55]. These practices, while often intended to fulfill clinical, logistical, or administrative needs, may inadvertently contribute to administrative discrimination. For instance, the use of the B20 diagnostic code for HIV/AIDS is based on the International Classification of Diseases (ICD) system and was originally implemented to facilitate accurate record-keeping, surveillance, and appropriate medical care [76]. Similarly, placing PLHIV in separate rooms may be driven by infection control protocols or concerns about protecting immunocompromised patients [77]. However, when these protocols are applied with exaggerated caution, such as visibly marking the B20 diagnosis code on the front page of medical records, a practice not commonly applied to other conditions, it may inadvertently offend PLHIV and reinforce stigma. This visible labeling can lead to unintended disclosure of HIV status and signals to both healthcare staff and other patients that the individual is “different”, fostering a discriminatory atmosphere within care settings. In addition, the use of isolation rooms, when accompanied by disproportionate protective measures such as excessive use of personal protective equipment or physical separation without clear medical indication, may further alienate PLHIV. Such measures, if not grounded in standard precautions recommended for all patients, risk reinforcing the perception that PLHIV is inherently dangerous or contagious, which is factually incorrect and counterproductive to stigma reduction efforts. Therefore, a comprehensive review of healthcare procedures should be considered. The procedures implemented should avoid including unnecessary measures that could potentially offend PLHIV. Furthermore, effective communication with patients can serve as a solution to address these issues.

In relation to the disclosure of HIV status among PLHIV, the identified literature reveals a consistent pattern in which stigma, whether in the form of marking or other manifestations, typically emerged once an individual’s HIV status became known. A similar pattern was also observed when individuals were identified as belonging to key populations or groups perceived to be at higher risk of HIV infection, such as family members of PLHIV. These findings suggest that disclosing one’s HIV status or being recognized as part of a key or high-risk population may in fact exacerbate the stigma experienced. Therefore, as long as HIV-related stigma remains a significant issue, disclosure of HIV status is likely to remain a sensitive and high-risk matter for PLHIV.

In addition to the external stigma previously discussed, self-stigma or internalized stigma remains a significant challenge in the context of HIV in Indonesia. Feelings of fear, shame, guilt, and sinfulness often haunt PLHIV, leading to a loss of motivation, social withdrawal, and reluctance to access healthcare services [9,48,51,58,59,60,61]. Therefore, stigma reduction efforts must include targeted interventions to address self-stigma. As a reinforcement of existing interventions, particularly the role of facilitators in reducing self-stigma among PLHIV, a more effective approach should not rely solely on one-on-one interactions between facilitators and PLHIV. It would be more optimal to complement these efforts with the establishment of peer support groups, which would enable PLHIV to share experiences, provide emotional support, and strengthen mutual solidarity. As an initial step, such peer support groups could be formed among PLHIV, who are registered for ARV at the same healthcare facility. Within these groups, educational sessions on HIV and related issues could also be conducted to enhance members’ knowledge and psychosocial resilience.

Although studies included in this review indicate that the percentage of HIV stigma remains high, it is important to note that most studies used data collected before 2019. This represents a limitation of the review, as it predominantly captures studies with older data, potentially overlooking more recent developments following public health interventions and awareness campaigns aimed at reducing stigma. This scoping review, when linked to The Health Stigma and Discrimination Framework, provides an overview of HIV stigma marking and manifestations in Indonesia. However, it lacks a comprehensive study of the driving factors, facilitator factors, effective interventions, and impacts of HIV stigma. Understanding these aspects is crucial for alignment with the framework and presents an opportunity for future research. Additionally, this review offers a preliminary understanding of HIV stigma in Indonesia. Future studies could adopt a systematic review approach to provide a more detailed and comprehensive picture, offering stronger evidence to guide interventions and policy recommendations.

## 5. Conclusions

HIV stigma continues to persist in Indonesia, manifesting in various forms. It occurs across multiple layers and sectors, including families, communities, students, adolescents, workplaces, healthcare workers, and even PLHIV themselves. These actions of stigma have significant consequences for affected groups, such as adult PLHIV, CLHIV, ALHIV, pregnant women, and key populations, leading to reduced access to healthcare services and adverse psychological effects, among other negative outcomes. The persistence of stigma is fueled by negative labeling and exaggerated fears among those perpetuating it. Therefore, efforts to intervene, including improving understanding of HIV among PLHIV, their families, communities, workplaces, and healthcare providers, might be crucial. Comprehensive interventions, such as collective action, might be essential for eradicating HIV stigma in Indonesia. In addition, the establishment of a strong regulatory framework might be critical to ensure equal opportunities and fair treatment for PLHIV, particularly in securing access to healthcare, social participation, and employment.

## Figures and Tables

**Table 1:** Literature characteristics.

Category	Sub-Category	Number (%)
Publication Year	2019	4 (10%)
	2020	7 (18%)
	2021	11 (28%)
	2022	13 (32%)
	2023	5 (12%)
Research Method	Qualitative	25 (63%)
	Quantitative	15 (37%)
Data Source Type	Primary	34 (85%)
	Secondary	6 (15%)
Research Locations ^1^	Ten provinces with the most PLHIV on ARV	16 (40%)
	Other provinces	15 (38%)
	General Indonesia	9 (23%)
Detail 10 provinces with the most PLHIV on ARV ^1^	DKI Jakarta	5 (12%)
	East Java	2 (5%)
	West Java	5 (12%)
	Central Java	0 (0%)
	Bali	3 (7%)
	Papua	3 (7%)
	North Sumatera	1 (2%)
	South Sumatera	2 (5%)
	Banten	1 (2%)
	Riau Island	0 (0%)
Study population ^2^	PLHIV	20 (50%)
	Key population	5 (13%)
	at-risk population	4 (10%)
	Students	3 (8%)
	General community	7 (18%)
	Healthcare workers	8 (20%)
	Other	2 (5%)
Findings ^3^	Manifestation	30 (75%)
	Marking	11 (27%)
Stigma score instrument	The AIDS-related stigma scale	1 (3%)
	Developed questionnaire	1 (3%)
	Nursing’ attitudes AIDS scale	1 (3%)
	No stigma score	37 (91%)
N = 40		

^1^ There were studies conducted in more than one location. ^2^ There were studies that involved more than one population group. ^3^ There were studies that contained more than one category of findings.

**Table 2:** Manifestations and markings of HIV stigma across different settings: family, community, healthcare workers, and workplace institutions.

Stigma Setting	Family	Comunnity	Healthcare Workers	Workplace Institutions
**Types of stigma manifestations**				
Avoiding contact				
Different treatment				
Negative social reactions				
**Types of stigma markings**				
Bad/dirty/immoral person				Not found
Dangerous person				

Grey background color means that the articles in the review contain types of stigma described.

## Data Availability

There are no data supporting the reported results in this study that have been uploaded to Google Drive.

## Supplementary Materials

The following supporting information can be downloaded at: https://drive.google.com/drive/folders/1ie_xYSrcTxG7fr-fPObbQSxjOH8bvJD0?usp=drive_link (accessed on 15 February 2025). File S1: RISMA-ScR-Fillable-Checklist_; File S2: Keywords for literature searching; File S3: List keyword for abstract and title screening; File S4: Literature included.
